# Targeted delivery of neural progenitor cell-derived extracellular vesicles for anti-inflammation after cerebral ischemia

**DOI:** 10.7150/thno.56367

**Published:** 2021-04-19

**Authors:** Tian Tian, Lei Cao, Chuan He, Qing Ye, Ruyu Liang, Weiyan You, Huixin Zhang, Jiahuan Wu, Jinhai Ye, Bakhos A. Tannous, Jun Gao

**Affiliations:** 1 Department of Neurobiology, Key Laboratory of Human Functional Genomics of Jiangsu, Nanjing Medical University, Nanjing, Jiangsu Province 211166, China.; 2 Department of Dermatology, The Affiliated Wuxi No.2 People's Hospital of Nanjing Medical University, Wuxi, Jiangsu Province 214002, China.; 3 Department of Rehabilitation Medicine, Jiangsu Shengze Hospital Affiliated to Nanjing Medical University, Suzhou, Jiangsu Province 215228, China.; 4 The Department of Oral and Maxillofacial Surgery, The Affiliated Stomatology Hospital of Nanjing Medical University, Nanjing, Jiangsu Province 210029, China.; 5 Experimental Therapeutics and Molecular Imaging Lab, Department of Neurology, Massachusetts General Hospital and Harvard Medical School, Boston, MA 02129, United States.

**Keywords:** extracellular vesicles, exosomes, anti-inflammation, targeted delivery, cerebral ischemia

## Abstract

Ischemic stroke remains a major cause of death, and anti-inflammatory strategies hold great promise for preventing major brain injury during reperfusion. In the past decade, stem cell-derived extracellular vesicles (EVs) have emerged as novel therapeutic effectors in immune modulation. However, the intravenous delivery of EVs into the ischemic brain remains a challenge due to poor targeting of unmodified EVs, and the costs of large-scale production of stem cell-derived EVs hinder their clinical application.

**Methods:** EVs were isolated from a human neural progenitor cell line, and their anti-inflammatory effects were verified *in vitro*. To attach targeting ligands onto EVs, we generated a recombinant fusion protein containing the arginine-glycine-aspartic acid (RGD)-4C peptide (ACDCRGDCFC) fused to the phosphatidylserine (PS)-binding domains of lactadherin (C1C2), which readily self-associates onto the EV membrane. Subsequently, in a middle cerebral artery occlusion (MCAO) mouse model, the RGD-C1C2-bound EVs (RGD-EV) were intravenously injected through the tail vein, followed by fluorescence imaging and assessment of proinflammatory cytokines expression and microglia activation.

**Results:** The neural progenitor cell-derived EVs showed intrinsic anti-inflammatory activity. The RGD-EV targeted the lesion region of the ischemic brain after intravenous administration, and resulted in a strong suppression of the inflammatory response. Furthermore, RNA sequencing revealed a set of 7 miRNAs packaged in the EVs inhibited MAPK, an inflammation related pathway.

**Conclusion:** These results point to a rapid and easy strategy to produce targeting EVs and suggest a potential therapeutic agent for ischemic stroke.

## Introduction

Ischemic stroke is the leading cause of mortality worldwide and is often responsible for acquired disability 1. Unfortunately, the only approved treatment for this type of stroke is recanalization, which is limited to a narrow therapeutic window (<4.5 h) 2. An extensive number of recent investigations have revealed that anti-inflammatory strategies hold a great promise for extending the therapeutic window and prevent major brain injury during reperfusion 3,4. Stem cell-derived extracellular vesicles (EVs), which mirror the functions of stem cells, have emerged as novel therapeutic effectors in immunosuppression 5,6.

EVs are released by cells in all living systems that hold cargos including proteins, coding and noncoding RNAs, and DNAs 7,8. As natural intercellular shuttles, EVs present several features, including low immunogenicity, biodegradability, the ability to encapsulate endogenous bioactive molecules, and the ability to cross the blood-brain barrier (BBB) 9,10. However, unmodified EVs rapidly accumulate in the organs of the reticuloendothelial system (RES) after intravenous administration 11,12. Integrin α_v_β_3_, being markedly expressed on endothelial cells during angiogenesis in abnormal conditions such as salvage of ischemic tissues and tumour progression but not on vessels in normal tissues, has been investigated intensively as target for diagnostic probes and delivery of therapeutics 13. Arg-Gly-Asp (RGD) peptides are well-known ligands bind to integrin α_v_β_3_ 14. RGD-based nanomaterials have been demonstrated success for delivering therapeutic or contrast agents to ischemic brain 15,16.

Various strategies have been employed to decorate ligands on EVs 17,18. For instance, ligand-displayed EVs were produced by engineering donor cells 19. However, this process is complex and cannot be readily applied to preisolated EVs 17. Additionally, we previously developed a chemical method to conjugate targeting peptides onto EV surfaces using click chemistry (azide alkyne cyclo-addition reaction) 20,21. This strategy allows ligand conjugation on preisolated EVs in 24 h. Recently, Kooijmans *et al*. reported an appealing strategy to decorate EVs with a recombinant fusion protein *via* affinity to phosphatidylserine (PS) on the EV membrane in a rapid 'plug-and-play' fashion 22. However, only a proof-of-principle experiment was performed, and *in vivo* conformation was lacking.

Another hurdle for the clinical translation of stem cell-derived EVs is the cost associated with their production, as large-scale EV isolation requires stem cell replenishment (limited expansion ability) and validation of each new batch 23,24. ReNcell VM (ReN) cells, a neural progenitor cell line derived from the ventral mesencephalon region of the human fetal brain, retain a normal diploid karyotype and differentiation capability in culture even after prolonged passage (>45 passages) 25,26. As ReN cells partially maintain the properties of stem cells 26,27, we hypothesize that ReN cell-derived EVs (EV_ReN_) may hold anti-inflammatory characteristics.

In this study, on lipopolysaccharide (LPS)-stimulated BV2 microglia, we showed that EV_ReN_ exhibited a distinct anti-inflammatory effect. Given that the C1 and C2 domains (together referred to as C1C2) of lactadherin (also named milk fat globule-EGF factor 8 protein, MFGE8) bind to PS 28,29, we generated a recombinant fusion protein of the RGD-4C peptide (ACDCRGDCFC) fused to C1C2 (RGD-C1C2) and demonstrate the association between RGD-C1C2 and EVs. In a middle cerebral artery occlusion (MCAO) and reperfusion (MCAO/R) mouse model, RGD-C1C2-decorated EV_ReN_ (RGD-EV_ReN_) were administered and then targeted the lesion region of the ischemic brain. Furthermore, we showed that poststroke inflammation was effectively suppressed by RGD-EV_ReN_. miRNA sequencing of EVs revealed a set of 7 miRNAs enriched in EV_ReN_ that suppressed mitogen-activated protein kinase (MAPK) signaling, an inflammation related pathway. Collectively, our data show that EV_ReN_ is a novel anti-inflammatory agent. Also, the incorporation of RGD-C1C2 onto EV_ReN_ can significantly improve their targeting ability and therapeutic efficacy for cerebral ischemia.

## Methods

### Cell culture

ReN cells were purchased from Millipore (Billerica, USA). The cells were cultured in DMEM/F12 (Life Technologies, Grand Island, USA) supplemented with 2% B27 (Life Technologies), 20 μg/mL EGF (Abm, Richmond, Canada), 10 μg/mL bFGF (Abm) in an incubator with 5% CO_2_ at 37 °C. BV2 and human embryonic kidney (HEK293T) cells (Type Culture Collection of the Chinese Academy of Sciences, Shanghai, China) were maintained in DMEM (Life Technologies) containing 10% fetal bovine serum (FBS) (PAN, Aidenbach, Germany) in a CO_2_ incubator. For HEK293T cell-derived EVs (EV_293_) isolation, EV-depleted FBS (prepared by overnight centrifugation at 200,000 g at 4 °C) was used. To label EVs with tdTomato, ReN cells were stably transduced with packaged lentivirus vectors to express tdTomato fused to palmitoylation signal (palm-tdTomato) which labels cell membrane 30. In addition, to display *Gaussia* luciferase (Gluc) on EV surface, lentivirus expressing Gluc fused to the transmembrane domain (TM) of the platelet-derived growth factor receptor (Gluc-TM) was packaged to stably transduce ReN cells 11. Both of Palm-tdTomato and Gluc-TM constructs have been verified in previous studies 11,30.

### EV isolation

ReN cells were cultured for 72 h and the supernatant was collected. The supernatant was centrifuged at 300 g for 10 min, 1200 g for 20 min, and 10,000 g for 30 min at 4 °C to remove cells and debris and then filtered using a 0.22-μm filter (Millipore). The filtrate was centrifuged at 200,000 g for 90 min at 4 °C in a Type Ti70 rotor using an L-80XP ultracentrifuge (Beckman Coulter, Brea, USA). The pellet was resuspended in PBS and ultracentrifuged again at 200,000 g for 90 min. The EV pellets were resuspended with double-0.22 μm-filtered PBS. The protein concentration was determined by BCA protein assay (Pierce, Rockford, IL, USA). Western blotting was performed with anti-Alix, anti-TSG101, and anti-Calnexin antibodies (Abcam, Cambridge, UK) to analyze EV markers and negative marker. For EV_293_ isolation, HEK293T cells were cultured in DMEM with 10% EV-depleted FBS for 72 h. The supernatant was collected and conducted to differential centrifugation as described above.

### Transmission electron microscopy (TEM) and nanoparticle tracking analysis (NTA)

EVs were observed by a Tecnai G2 transmission electron microscope (FEI, Hillsboro, USA). Samples were fixed with 1% glutaraldehyde, applied onto a carbon-coated copper grid, and stained with 1% phosphotungstic acid. NTA was performed using a ZetaView system (Particle Metrix, Meerbusch, Germany) to track the Brownian motion of EVs suspended in PBS, and size distribution data was generated by applying the Stokes-Einstein equation.

### Plasmid construct and virus package

The RGD-4C peptide was designed to fuse with the C1C2 region of lactadherin with a 2×GGGGS linker sequence. The signal sequence (SS) of Gluc was inserted to induce protein secretion. The designed SS-RGD-linker-C1C2-HA-His_6_ sequence flanked with the NheI site was synthesized by GenScript (Nanjing, China) and cloned into pCSCW-IG, a lentivirus gene-transfer plasmid that expresses GFP separately by an internal ribosome entry site (IRES) element under the control of the cytomegalovirus (CMV) promoter (pCSCW-CMV-SS-RGD-linker-C1C2-HA-His_6_-IRES-GFP). As a control, a scrambled ACDCRDGCFC was synthesized with SS and linker-C1C2-HA-His_6_ and then cloned into pCSCW-IG. Lentivirus was generated by transient transfection of HEK293T cells with the packaging plasmid pCMVΔ8.91, the envelope-plasmid pVSV-G, and the gene-transfer-plasmids. Forty-eight hours after transfection, virus particles were harvested, concentrated by ultracentrifugation, and stored at -80 °C.

### Preparation of recombinant proteins and EV decoration

HEK293T cells were stably transduced with packaged lentivirus to express RGD-C1C2 or a scrambled ACDCRDGCFC peptide fused to C1C2 (Scr-C1C2). Cells were cultured for 48 h, and the conditional medium was harvested. After depletion of cells and debris by centrifugation at 10,000 g for 20 min at 4 °C, the supernatant was concentrated using a 10-kD ultrafiltration tube (Millipore). The concentrate was incubated with 0.5% Triton X-100 for 30 min to disrupt protein-EV interactions. RGD-C1C2 or Scr-C1C2 was purified using a His-tag Protein Purification Kit (Beyotime, Shanghai, China) according to the manufacturer's instructions and quantified by BCA protein assay (Pierce). Western blot analysis was performed using an anti-HA antibody (Sigma, St. Louis, USA) to detect recombinant RGD-C1C2. For EV decoration, 0.5 mg/mL EVs were incubated with 1 μg/mL RGD-C1C2 or Scr-C1C2 for 15 min at room temperature (RT). Unbound proteins were removed by ultracentrifugation at 200,000 g for 90 min at 4 °C.

### EV pull-down assay

tdTomato-labeled or Gluc-displayed EVs were decorated with RGD-C1C2 or not. Ten microgram (2.3-3.6 × 10^9^) EVs or RGD-EV were incubated with 50 μg anti-HA magnetic beads (SinoBiological, Beijing, China) on a rotating mixer for 2 h at RT. For the blocking experiments, the EVs were preincubated with 5 μg/mL lactadherin for 15 min prior to RGD-C1C2 adding, or the beads were preincubated with 20 μg/mL HA peptide or the scrambled YVYPDAYPD peptide for 30 min prior to EV adding. Then the magnetic beads were collected with a magnetic separator, washed and resuspended by PBS. The tdTomato-labeled EVs associated with the beads were imaged by a Ti-E fluorescence microscope (Nikon, Tokyo, Japan) using a 100× objective (NA. = 1.40). For Gluc signal detection, the beads were added into a white 96-well plate and measured by a GloMax luminometer (Promage, Fitchburg, USA) with automated injection of 50 μL of 10 ng/mL coelenterazine (CTZ, Nanolight, Pinetop, USA) followed by photon counts for 10 s.

### EV uptake analysis

To investigate the uptake of RGD-EV by BV2 cells, the cells were stained by carboxyfluorescein diacetate, succinimidyl ester (CFSE, Sigma) and incubated with tdTomato-labeled RGD-EV. Cofocal imaging was performed 2 h later. For quantitative analysis of the enhanced uptake of RGD-EV, Gluc-displayed EVs were isolated and decorated by RGD-C1C2 or Scr-C1C2. BV2 cells were incubated with 30 μg/mL (6.6-9.5 × 10^9^/mL) Gluc-displayed EVs, scrambled peptide-decorated EVs (Scr-EV), or RGD-EV for 2 h at 37 °C. Subsequently, the cells were washed by PBS, lysed by 200 μL Passive Lysis Buffer (Promega), and centrifuged at 10,000 g for 10 min. Fifty microliters of the supernatant was plated in triplicates into a white 96-well plate. Gluc activity was measured by a GloMax luminometer (Promage) with automated injection of 50 μL of 10 ng/mL CTZ (Nanolight) followed by photon counts for 10 s.

### Mice and cerebral ischemia model

C57BL/6 mice (8 weeks old) were provided by the Animal Core Facility of Nanjing Medical University (Nanjing, China). All animal experiments were carried out in compliance with institutional guidelines and were approved by the Animal Care and Use Committee of Nanjing Medical University. For transient focal cerebral ischemia, mice were subjected to MCAO as described before 20. Briefly, the right middle carotid artery (MCA) was occluded by inserting a 6-0 nylon monofilament suture into the right internal carotid artery. Reperfusion was allowed by suture removal 1 h after occlusion. A 75-90% blood flow decrease in the MCA territory was recorded by laser Doppler flow during each experiment after reperfusion using a flexible probe attached to the animals' skull. In the sham group, an identical surgical procedure was performed without disturbing the arteries. Twenty-four hours after reperfusion, the brains were dissected, sliced, and stained by 0.25% 2,3,5-Triphenyltetrazolium chloride (TTC) dye. The pallor area indicated ischemic infarct, and the lesion region was demarcated according to previous reports 20,31.

### Near-infrared fluorescence (NIRF) imaging

To label EVs with near-infrared fluorophore, 500 μg/mL EVs were incubated with 0.3 μM Cy5.5 NHS ester (Lumiprobe, Hallandale Beach, USA) on a rotating mixer for 4 h at RT. Then, the EVs were floated on a 30% sucrose/D_2_O cushion and centrifuged at 164,000 g for 90 min using an SW41Ti rotor (Beckman Coulter) to remove unbound fluorophore, and washed with PBS by ultracentrifugation. After decoration by RGD-C1C2 or Scr-C1C2, 100 μg (2.5-3.7 × 10^10^) Cy5.5-labeled EVs, Scr-EV, or RGD-EV were intravenously administered 12 h after reperfusion in mouse MCAO/R model. Twenty-four hours after administration, the mice were anesthetized and sacrificed. The brain, heart, lungs, liver, spleen, and kidneys were dissected and imaged by an IVIS Spectrum imaging system (PerkinElmer, Waltham, USA). The Cy5.5-related fluorescence signals were discriminated from the auto-fluorescence signals using Living Image software (PerkinElmer).

### Quantitative real-time PCR (RT-PCR) and enzyme-linked immunosorbent assay (ELISA) for proinflammatory cytokines assessment

BV2 microglia were treated with PBS, 10-40 μg/mL (2.2 × 10^9^ - 1.3 × 10^10^/mL) EV_293_ or EV_ReN_ for 24 h and then 1 μg/mL LPS were added. The cells were harvested 1 h later for RT-PCR or 24 h later for ELISA. For the *in vivo* experiments, 300 μg (6.7-9.2 × 10^10^) unmodified EVs, Scr-EV, or RGD-EV were suspended in 0.2 mL PBS and administered *via* a single intravenous injection into the tail vein 12 h after reperfusion. The tissues corresponding to the lesion region were then dissected 12 h after administration for RT-PCR or 24 h after administration for ELISA.

For RT-PCR, total RNA of the cells or tissues was extracted by Trizol reagent (Invitrogen). cDNA synthesis was performed using a PrimeScript RT reagent Kit (Takara). PCR reactions were carried out by a Lightcycler 96 system (Roche) in 20 μL reactions, with 2 μL cDNA samples, using SYBR Mix (Vazyme, Nanjing China). Relative expression was calculated by the comparative 2^-ΔΔCt^ method. All experiments were performed at least three times independently. The primers used were as follows: (forward) 5'-GACAGTGACCTGGACTGTGG-3' and (reverse) 5'-TGAGACAGAGGCAACCTGAC-3' for tumor necrosis factor-α (TNF-α); (forward) 5'-TCAGGCAGGCAGTATCACTC-3' and (reverse) 5'-TCATCTCGGAGCCTGTAGTG-3' for interleukin-1β (IL-1β); (forward) 5'-CCAATTTCCAATGCTCTCCT-3' and (reverse) 5'-ACCACAGTGAGGAATGTCCA-3' for interleukin-6 (IL-6); (forward) 5'-GGCTGTATTCCCCTCCATCG-3' and (reverse) 5'-CCAGTTGGTAACAATGCCATGT-3' for β-actin.

For ELISA, the conditional medium of BV2 cells was centrifuged to remove cells and subjected to TNFα, IL-1β, or IL-6 ELISA Kits (MultiSciences, Hangzhou, China). The tissue samples were homogenized with PBS and centrifuged at 12,000 g for 20 min. The concentrations of TNFα, IL-1β, and IL-6 were determined by ELISA Kits according to the manufacturer's protocols (MultiSciences). Readings from each sample were normalized to the medium volume or the protein concentration.

### RT-PCR for miRNA evaluation

Total RNA was extracted from cells and EVs using Trizol reagent. For EV RNA extraction, 100 μg EVs were used with 10 femto-moles cel-miR-39 as an external reference and an RNeasy MinElute Spin Column (Qiagen, Hilden, Germany) was employed. To detect miRNAs, miRcute Plus miRNA First-Strand cDNA Kit, qPCR Kit and primers (TIANGEN, Beijing, China) were used following the manufacturer's protocol. Relative expression was calculated by the comparative 2^-ΔΔCt^ method.

### Immunofluorescence staining and confocal imaging

For immunofluorescence assay, 100 μg (2.3-3.5 × 10^10^) tdTomato-labled EV_ReN_, scrambled peptide-decorated EV_ReN_ (Scr-EV_ReN_), or RGD-EV_ReN_ were intravenously administered 12 h after reperfusion. Six hours later, the mice were perfused with 25 mL PBS and 25 mL 4% paraformaldehyde (PFA). The brains were removed, kept in 4% PFA overnight and 30% sucrose for 48 h, and then cryosectioned in 40-μm thickness. The sections were treated by 0.3% Triton X-100 for 30 min and by 3% BSA for 2 h, and then stained with anti-integrin β_3_ (Santa Cruz) or anti-CD34 (Abcam) overnight at 4 °C. After 5 washes with PBST (PBS containing 0.1% Triton X-100), the samples were incubated with Alexa 488- or Alexa 647-conjugated secondary antibodies (Invitrogen) for 1 h at RT. After another 5 washes with PBST, staining with Hoechst 33342, and mounting with ProLong Antifade Reagents (Invitrogen), the slides were imaged with an FV-1200 confocal microscope (Olympus, Tokyo, Japan). For assessed the activation of microglia, immunofluorescence staining was performed 24 h after administration with anti-ionized calcium binding adapter molecule 1 (Iba-1) antibody (Wako, Osaka, Japan). Images were processed and analyzed using ImageJ software (NIH, Bethesda, USA). All settings of imaging and processing were kept constant. The fluorescence intensities of Iba1 per cell were collected from six random imaging fields for each independent experiment and averaged.

### miRNA sequencing and analysis

For miRNA sequencing, RNA was isolated by Trizol (Life Technologies) extraction from EV_ReN_ and EV_293_. Qubit 2.0 and Agilent 2100 bioanalyzer were used to quantify the samples. The cDNA libraries were produced using a NEBNext Ultra small RNA Sample Library Prep Kit for Illumina according to the manufacturer's instructions. Subsequently, the library preparations were sequenced on an Illumina Hiseq 2500 platform and paired-end reads were generated. Using Bowtie software, the clean reads were compared with Silva database, GtRNAdb database, Rfam database and Repbase database sequence alignment to filter ribosomal RNA (rRNA), transfer RNA (tRNA), small nuclear RNA (snRNA), small nucleolar RNA (snoRNA) and other ncRNA and repeats. The remaining reads were used to detect known miRNA and new miRNA predicted by comparing with known miRNAs from miRBase. The miRNA levels were calculated and normalized to transcripts per million (TPM). A heatmap analysis of miRNA expression levels was created based on the TPM values of miRNAs in EV_ReN_ and EV_293_ (using TPM_average_ > 1000, 1.5-fold change and *p* < 0.05 as the threshold cutoff). Kyoto Encyclopedia of Genes and Genomes (KEGG) analyses were performed to determine enriched pathways involved in the predicted target genes of differentially expressed miRNAs.

### Statistical analysis

The data are presented as mean ± SEM of at least three independent experiments (the n were included in the figure legends). For RT-PCR, Western blots, bioluminescence assays, and NIRF images, three technical replicates were collected from each independent experiment to account for variability in the sample. For ELISA, six technical replicates were collected and averaged. For assessment of microglia activation, the fluorescence intensities of Iba1 per cell were collected from six random imaging fields for each independent experiment and averaged. Statistical analysis was accomplished using GraphPad Prim software (GraphPad Software, San Diego, CA, USA). Comparison between two groups was performed by Student's *t*-test. The significances among multiple groups were determined by One-way Analysis of Variance (ANOVA) followed by Bonferroni *post hoc* test. *P* value < 0.05 was considered statistically significant.

## Results

### The anti-inflammatory activity of EV_ReN_ *in vitro*

**Figure 1:**
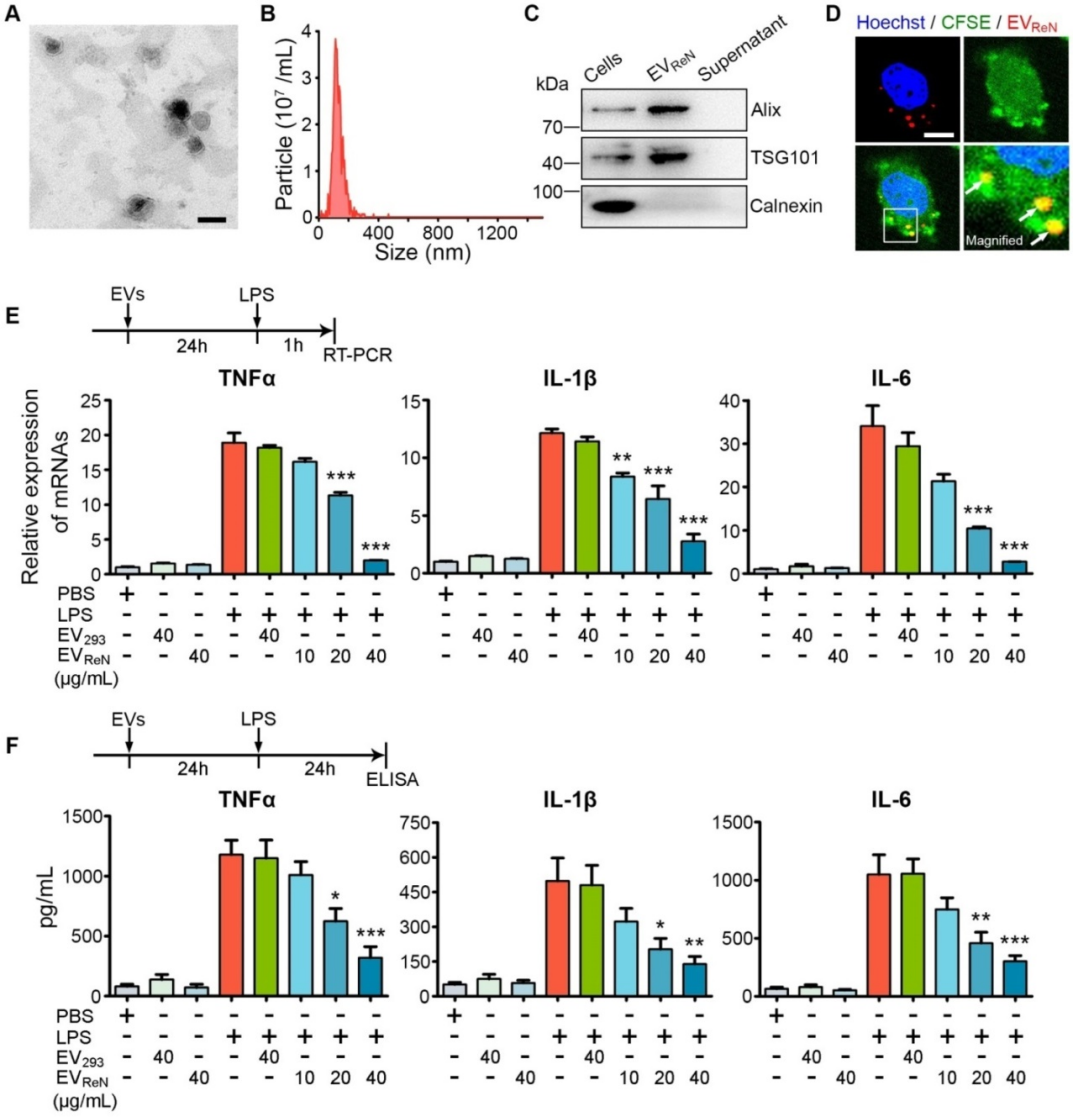
**Characterization of ReN cell-derived EVs.** (A) Transmission electron micrograph of EV_ReN_. Scale bar, 100 nm. (B) Size distributions of EV_ReN_ based on NTA measurements. (C) Western blot analysis of Alix, TSG101, and Calnexin from ReN cells and EVs isolated from their conditioned medium. The supernatant obtained from the ultracentrifugation during EV isolation was used as a negative control. (D) Representative confocal images of cellular uptake of tdTomato-labeled EV_ReN_ after 2-h incubation with BV2 microglia. Red shows EVs. Green indicates cell profile. Blue is nuclei. Arrows indicate the EVs in cytoplasma. Scale bar, 30 µm. (E, F) LPS-stimulation was performed on BV2 microglia receiving 24 h of PBS, EV_293_, or EV_ReN_ treatments. (E) RT-PCR analyses (n = 4) of TNF-α, IL-1β, and IL-6 in the cells were performed 1 h after LPS-induction. (F) ELISA analyses (n = 5) of TNF-α, IL-1β, and IL-6 in the conditioned medium were performed 24 h after LPS-induction. Data are expressed as mean ± SEM. *P < 0.05, **P < 0.01, ***P < 0.001 versus the LPS-stimulation without treatment groups by One-way ANOVA.

Human neural progenitor ReN cells were cultured in serum-free medium for 3 days (**Figure S1**). The conditioned medium was collected and EVs were isolated by ultracentrifugation. The typical EV protein yield was 25-35 μg per mL of culture medium, corresponding to 5.5 ×10^9^ - 8 × 10^9^ particles per mL of culture medium as determined by NTA. The morphology and size distribution of EVs were confirmed by TEM and NTA (**Figure 1A-B**). Western blot analysis showed the enrichment of EV markers (Alix and TSG101) in EV pellets, whereas calnexin (a typical negative marker) was not detected (**Figure 1C**). We then labeled EVs with tdTomato by first engineering ReN cells with a lentivirus vector to express palm-tdTomato which labels cell membrane and thus EV membrane as described 30. BV2 microglia were stained by CFSE and then incubated with tdTomato-labeled EVs for 2 h. By cofocal imaging, EVs were observed in the BV2 cells, which showed cellular uptake of EVs (**Figure 1D**).

To evaluate the anti-inflammatory activity of EV_ReN_, mouse BV2 microglia were incubated with these EVs for 24 h prior to LPS stimulation and proinflammatory cytokines were detected using RT-PCR and ELISA. As a control, HEK293T cells were cultured with EV-depleted FBS (verified by NTA, as shown in **Figure S2**) and the EV_293_ were isolation. We observed that BV2 cells induced by 1 μg/mL LPS produced much more TNF-α, IL-1β, and IL-6 than untreated cells (**Figure 1E-F**). Treatment with EV_ReN_ resulted in significantly less proinflammatory cytokine production by activated BV2 cells than treatment with EV_293_. In addition, the anti-inflammatory efficacy of EV_ReN_ was dose-dependent. These results show that EV_ReN_ harness intrinsic anti-inflammatory activity.

### Preparation and characterization of targeted RGD-EV_ReN_

**Figure 2:**
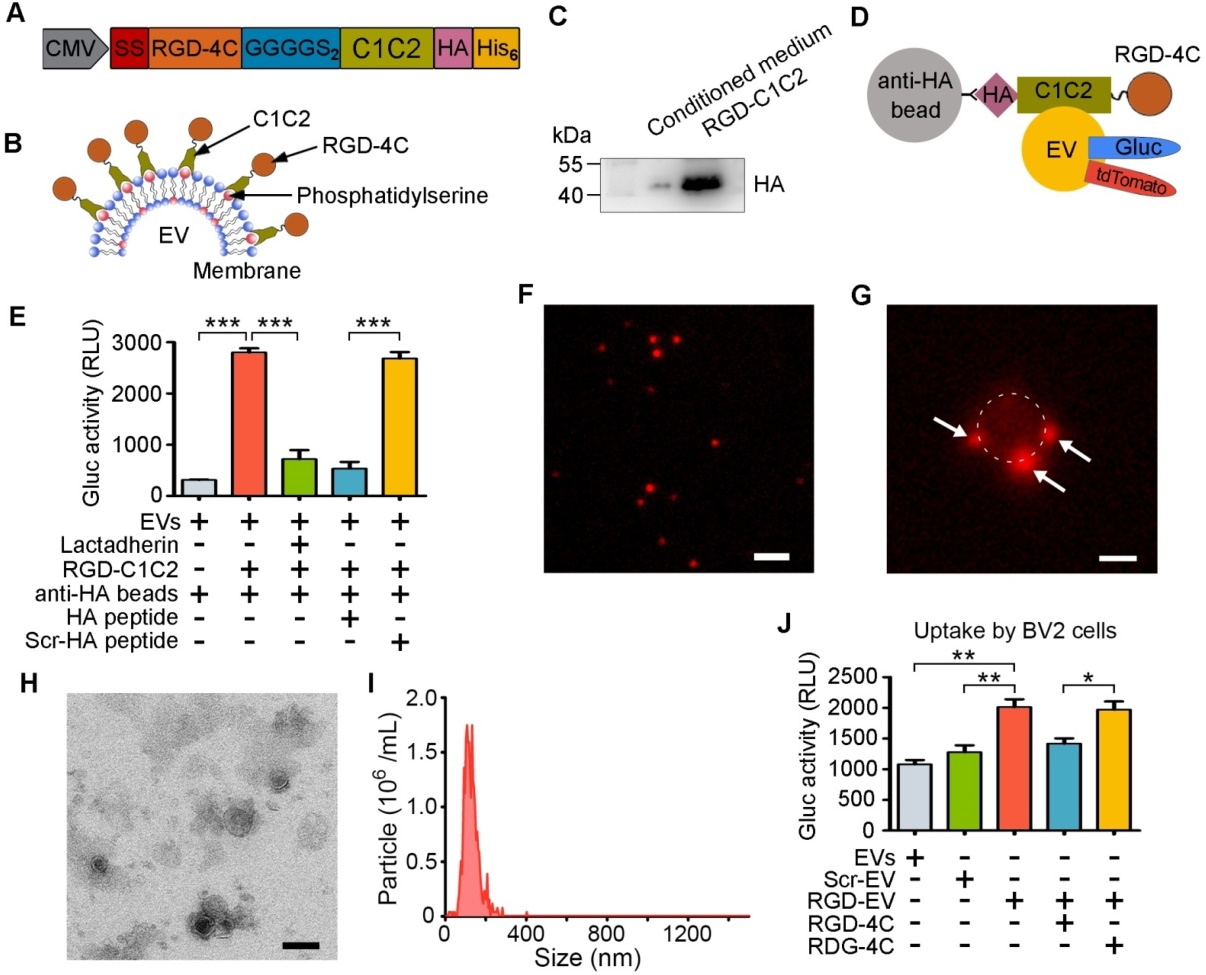
**Preparation and characterization of RGD-EV_ReN_.** (A, B) Schematic diagram of designing RGD-C1C2 and decorating EVs with RGD-C1C2. (A) Recombiant protein consists of a signal sequence (SS) for secreting, RGD-4C peptide sequence, 2×GGGGS linker sequence, C1C2 domains of lactadherin, and HA and His_6_ tags. (B) C1C2 domains of the recombiant protein bind phosphatidylserine on EV surface, resulting in decoration of RGD-4C. (C) Western blots of purified RGD-C1C2 and corresponding conditioned medium, stained by anti-HA antibody. (D) Schematic diagram of EV pull-down assay. tdTomato or Gluc-labeled RGD-EV_ReN_ are expected to be pulled-down by anti-HA beads through affinity to HA-tag on RGD-C1C2. (E) Anti-HA beads were applied to pull-down Gluc-displayed EV_ReN_ with RGD-C1C2-association or not followed by luminescence detection (n = 4). For blocking assays, EV_ReN_ were preincubated with lactadherin, or the beads were preincubated with the HA peptide or the scrambled HA peptide (Scr-HA). (F) tdTomato-labeled RGD-EV_ReN_ present small particles (Red) on fluorescent image. Scale bar, 2 µm. (G) RGD-EV_ReN_ (Red particles) appear on the beads after being pulled-down. The bead is around by dashed line. Scale bar, 1 µm. (H) TEM image of RGD-EV_ReN_. Scale bar, 100 nm. (I) Size distributions of RGD-EV_ReN_ based on NTA measurements. (J) Luminescence detection of cellular uptake (n = 4) of Gluc-displayed EV_ReN_ or Scr-EV_ReN_ or RGD-EV_ReN_ after 2-h incubation with BV2 microglia. For blocking assays, cells were preincubated with the RGD-4C peptide or the scrambled peptide (RDG-4C). All the quantitative data are expressed as mean ± SEM. **P < 0.01, ***P < 0.001 by One-way ANOVA.

To improve the targeting ability of EV_ReN_ to the lesion region in the ischemic brain, we conjugated the RGD-4C peptide to their surface. DNA sequences of these peptides were fused to PS-binding C1C2 domains of lactadherin with a 2×GGGGS linker sequence (**Figure 2A**). The signal sequence of the naturally secreted Gluc was inserted at the N-terminus to induce protein secretion, while HA and His_6_ tags were inserted at the C-terminus for detection and purification. EVs are more enriched in PS than their donor cells and do not contain flippase to restrict PS in the inner leaflet of the membrane 32. The recombinant fusion protein was hypothesized to self-associate with PS on the EV membrane, resulting in peptide decoration on the EV surface (**Figure 2B**). Next, HEK293T cells were stably transduced with a lentivirus to express RGD-C1C2. The protein was purified from the condition medium by His-tag affinity and appeared as bands near its calculated molecular weight of 39.72 kDa by Western blotting (**Figure 2C**).

For visualization and quantification of EVs, ReN cells were stably transduced to express palm-tdTomato or Gluc-TM to produce tdTomato- or Gluc-labeled EVs 11,30. The labeled EVs were incubated with RGD-C1C2 (with HA tag) and unassociated proteins were removed by ultracentrifugation. The linear relationship between bioluminescent signal of Gluc-labeled RGD-EV and the EV number was confirmed (**Figure S3**). Subsequently, anti-HA magnetic beads were employed pull down these EVs (**Figure 2D**). A luminometer was used to assess the EVs pulled down with the beads. The bioluminescent signals of RGD-C1C2-decorated EVs associated with anti-HA beads were significantly higher than those of undecorated EVs, and this signal was inhibited by preincubating EVs with lactadherin (containing C1C2 but no HA tag) or preincubating the beads with the HA peptide (**Figure 2E**). Furthermore, fluorescence microscopy analysis showed that the tdTomato-labeled RGD-EV_ReN_ presented as small particles (**Figure 2F**). After being pulled down, small particles appeared on the beads, suggesting that RGD-C1C2 was associated with the EVs (**Figure 2G**). Whereas, preincubation of the EVs with lactadherin or preincubation of the beads with the HA peptide prevented this association (**Figure S4**). These results indicate that the recombinant C1C2-containing proteins readily associated with EVs upon incubation together.

Next, TEM and NTA revealed that the morphology and size distribution of RGD-EV_ReN_ were unaltered and similar to undecorated EVs (**Figure 2H-I**). To investigate the tropism of RGD-EV_ReN_ *in vitro*, their uptake was assessed in BV2 cells expressing integrin α_v_β_3_. Scr-C1C2 was used as a negative control. Gluc activity in cells was detected to analyze the level of internalization. The uptake level of RGD-EV_ReN_ was significantly higher than that of undecorated EVs or Scr-EV_ReN_, and this uptake was inhibited by preincubating the cells with RGD-4C peptide (**Figure 2J**). These results indicate that the RGD-C1C2 effectively attaches the RGD-4C peptide onto the EV surface and that the resulting RGD-EV_ReN_ exhibited an affinity for cells expressing integrin α_v_β_3_.

### The ability of RGD-EV_ReN_ to target ischemic brain regions

**Figure 3:**
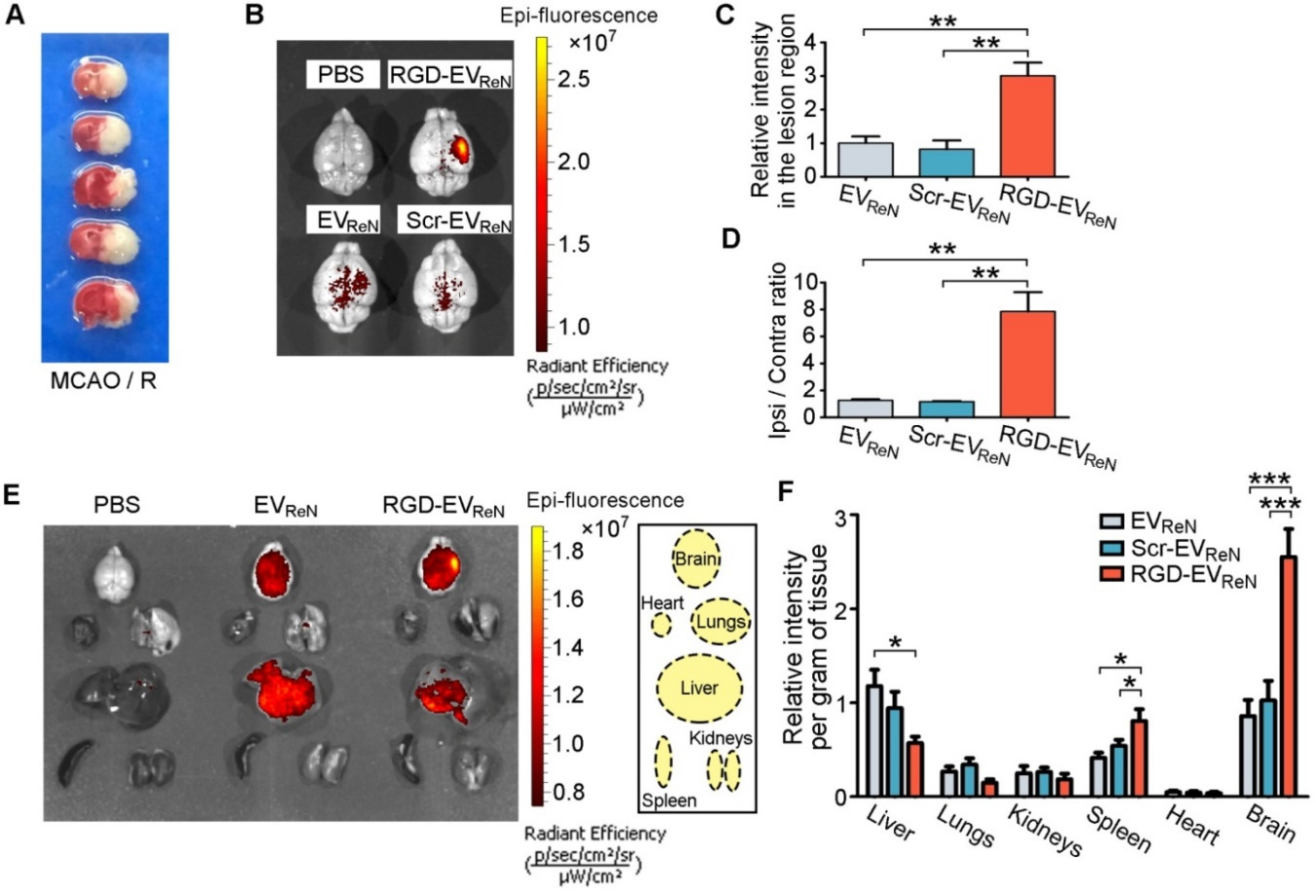
** Ability of RGD-EV_ReN_ to target the lesion region of ischemic brain**. (A) TTC-stained brain sections show the typical infarct area in a mouse receiving 1 h of MCAO and 24 h of reperfusion. (B) Representative NIRF images (overlaid with photograph) of mice brains which received MCAO/R and the administration of PBS, Cy5.5-labeled EV_ReN_, Scr-EV_ReN_ or RGD-EV_ReN_. Brains were dissected 24 h after administration (36 h after reperfusion). (C) Quantitation of fluorescence intensity (n = 5) in the lesion region. (D) Ratios of fluorescence intensity (n = 5) in ipsilateral versus contralateral region. Data in C and D are expressed as mean ± SEM. **P < 0.01 by One-way ANOVA. (E) Representative NIRF images of organs dissected from mice which received MCAO/R along with the administration of PBS, Cy5.5-labeled EV_ReN_ or RGD-EV_ReN_. Organs were dissected 24 h after administration (36 h after reperfusion). Right boxed graph illustrates location of the analyzed six organs. (F) Quantitation of fluorescence intensities (n = 6) per gram of tissue in different organs. Data are expressed as mean ± SEM. *P < 0.05, ***P < 0.001 by One-way ANOVA.

To evaluate the targeting ability of RGD-EV_ReN_ to ischemic brain regions, mice were subjected to MCAO/R in the right hemisphere. TTC staining was applied to confirm the infarct areas (**Figure 3A**). In this well-established animal model, the lesion region is demarcated according to previous reports 31,33, and the inflammatory response peaks at 18-24 h after reperfusion 34. Herein, Cy5.5-labeled EVs were decorated with RGD-C1C2 or Scr-C1C2 or not and then intravenously injected into mice (100 μg total protein, approximate 2.5-3.7 × 10^10^ EVs per mouse) that received 1 h of MCAO and 12 h of reperfusion. Twenty-four hours later, the brains were dissected and analyzed by NIRF imaging. Cy5.5 fluorescence was remarkably aggregated in the lesion region after administration of RGD-EV_ReN_, whereas the unmodified EV_ReN_ or Scr-EV_ReN_ produced much less fluorescence (**Figure 3B-C**). Notably, the fluorescence ratio of the lesion region (ipsilateral) to the nonischemic region (contralateral) increased dramatically to as high as 7.84 following the injection of RGD-EV_ReN_ (**Figure 3D**). These data indicate that RGD-4C peptide decoration significantly enhanced the tropism of EVs to the lesion region of the ischemic brain. Biodistribution analysis of EVs in different organs by NIRF imaging revealed that the accumulation of undecorated EV_ReN_ or Scr-EV_ReN_ was most predominant in the liver, followed by the ischemic brain and then the spleens and lungs, whereas the RGD-EV_ReN_ had a significantly stronger signal in the ischemic brain (**Figure 3E-F, Figure S5**).

**Figure 4:**
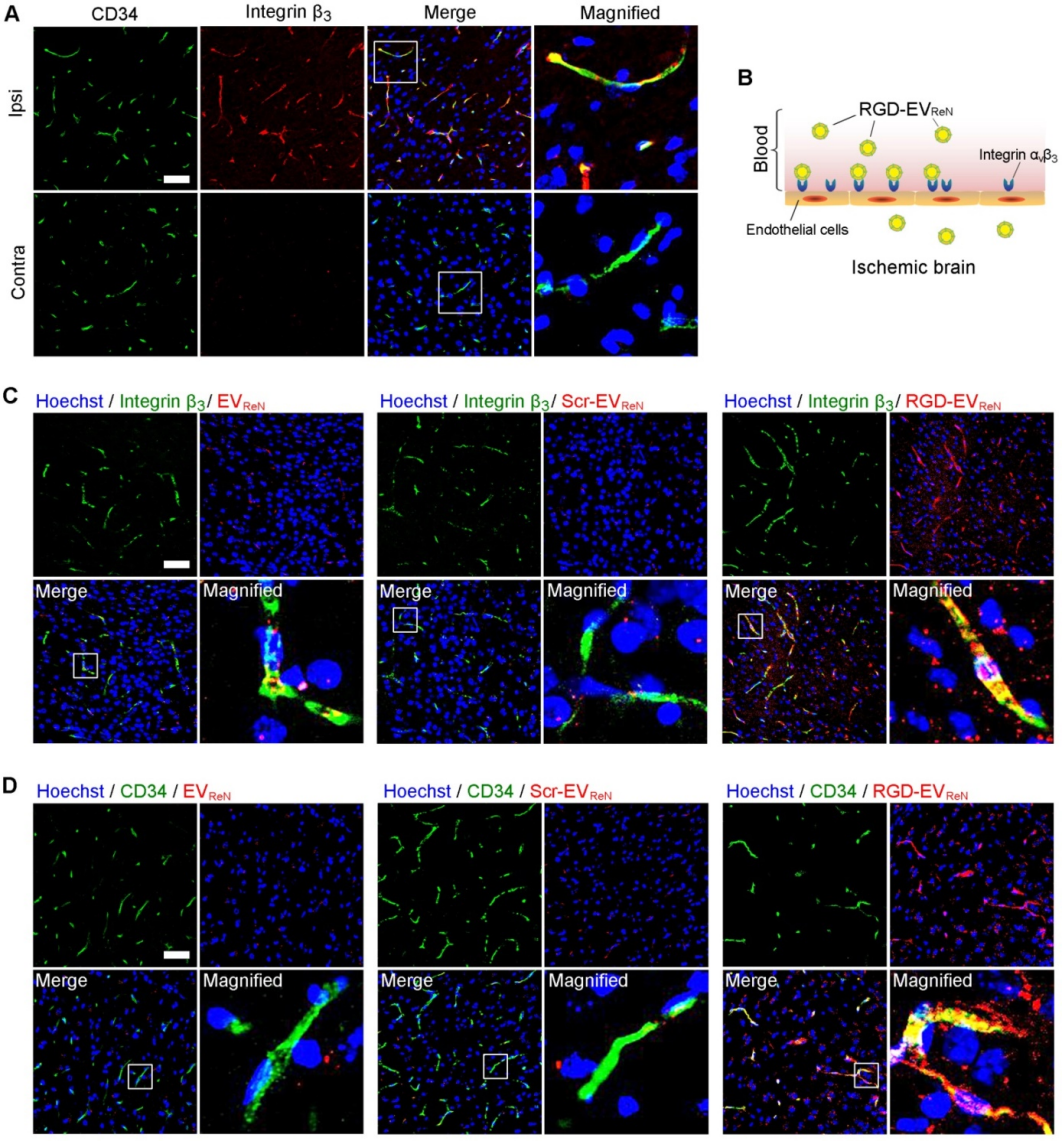
** Recognition of integrin β_3_ on reactive endothelial cells by RGD-EV_ReN_.** (A) Co-labeled fluorescence images of vascular endothelial cells (CD34, green) with integrin β_3_ (red) in the lesion region (ipsilateral) and non-ischemic region (contralateral) 12 h after reperfusion. (B) Schematic diagram of RGD-EV_ReN_ binding integrin α_v_β_3_ on reactive endothelial cells in ischemic brain after intravenous administration. (C,D) Co-labeled fluorescence images of integrin β_3_ or CD34 (green) with EV_ReN_, Scr-EV_ReN_, or RGD-EV_ReN_ (red) in the lesion region 6 h after intravenous administration (18 h after reperfusion). Blue indicates nuclei. Scale bars, 50 µm.

To understand the targeting ability of RGD-EV_ReN_ at the histological level, the brains were sectioned after 1 h of MCAO and 12 h of reperfusion. Integrin β_3_, which exclusively partners with integrin α_v_ on angiogenic brain endothelial cells 35, was immunostained and observed by confocal microscopy. Integrin β_3_ appeared in CD34-marked vascular endothelial cells in the lesion region (ipsilateral) (**Figure 4A**). Conversely, minimal overlap between integrin β_3_ and CD34 was observed in the nonischemic region (contralateral). These results are consistent with the literature on integrin α_v_β_3_ expression in reactive vascular endothelial cells induced by ischemia 36,37. Thus, we hypothesized that RGD-EV_ReN_ binds to integrin α_v_β_3_ on vascular endothelial cells in the ischemic brain after intravenous administration (**Figure 4B**). To test this hypothesis, tdTomato-labeled EV_ReN_, Scr-EV_ReN_, or RGD-EV_ReN_ were intravenously injected into mice who received 1 h of MCAO and 12 h of reperfusion. After 6 h of blood circulation, the brains were sectioned and examined by immunostaining which showed some RGD-EV_ReN_ localized in integrin β_3_-positive vessels (**Figure 4C-D**). In contrast, the overlap between EV_ReN_/Scr-EV_ReN_ and integrin β_3_/CD34 was minimal. Interestingly, some RGD-EV_ReN_ did not colocalized with vascular endothelial cells but appeared in brain parenchyma. To observe RGD-EV_ReN_ at an earlier time point, we sectioned the brain tissue 30 min after intravenous administration. Immunostaining showed a significant portion of RGD-EV_ReN_ were in the CD34-marked vessels (**Figure S6**). These results suggest that RGD-EV_ReN_ may binds to integrin α_v_β_3_ on endothelial cells, and enter brain parenchyma in the lesion region. Moreover, tdTomato-labeled EV_ReN_ or RGD-EV_ReN_ in the livers, lungs, kidneys, spleens and hearts 24 h after administration were imaged (**Figure S7**). Taken together, the signals in the livers and brains were observed in the tissue sections.

### The suppression of the postischemia inflammatory response by RGD-EV_ReN_

**Figure 5:**
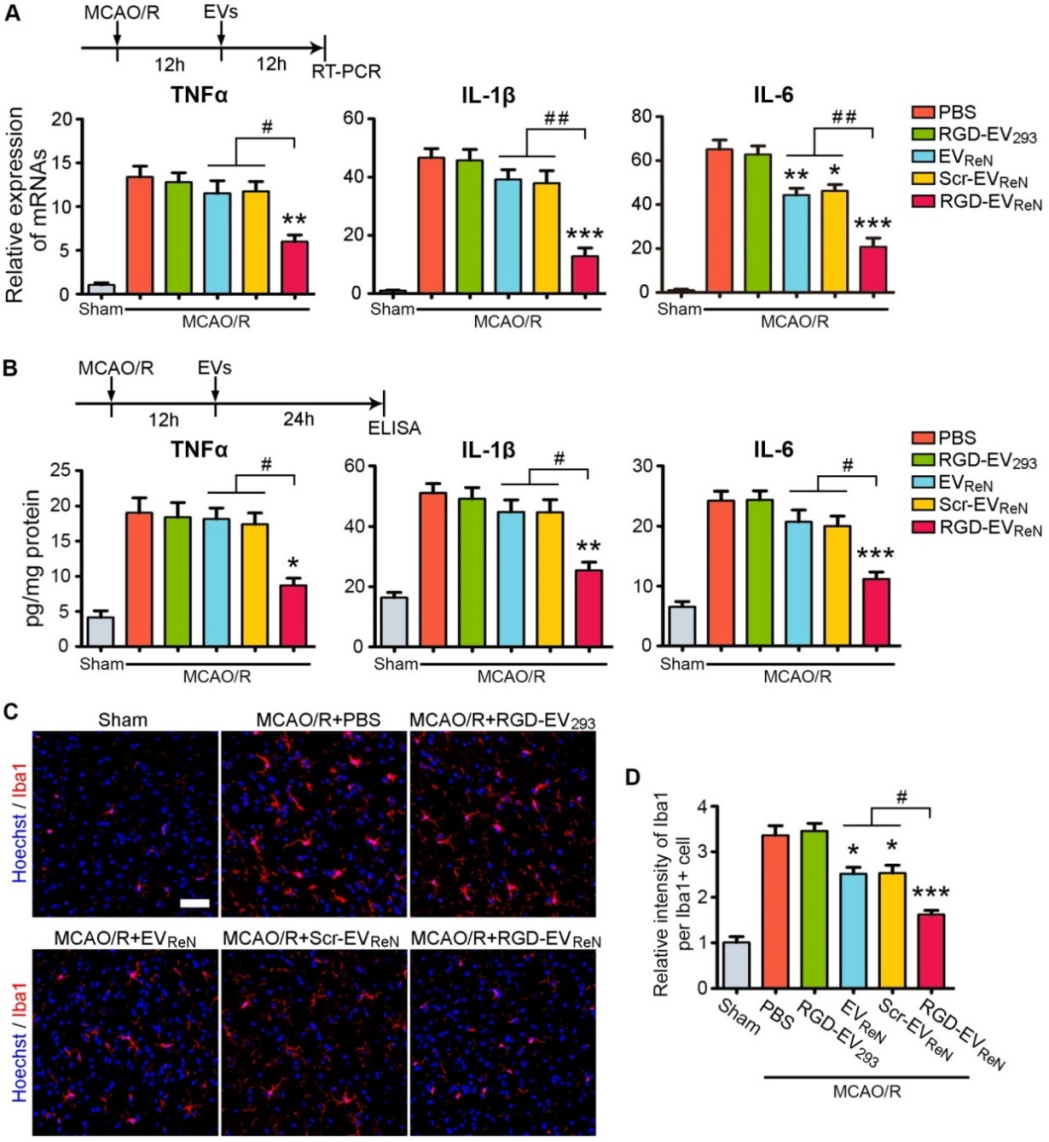
** The inhibitory effect of RGD-EV_ReN_ on inflammatory response after cerebral ischemia.** Intravenous administrations of PBS, RGD-4C-decorated EV_293_ (RGD-EV_293_), EV_ReN_, scrambled peptide-decorated EV_ReN_ (Scr-EV_ReN_), or RGD-4C-decorated EV_ReN_ (RGD-EV_ReN_) were performed on mice receiving 1 h of MCAO and 12 h of reperfusion. (A) RT-PCR analyses (n = 5) of TNF-α, IL-1β, and IL-6 in the tissue of lesion region were performed 12 h after administration (24 h after reperfusion). (B) ELISA analyses (n = 5) of TNF-α, IL-1β, and IL-6 in the supernatant of homogenized lesion tissue were performed 24 h after administration (36 h after reperfusion). (C) Representative fluorescence images of Iba1-stained microglia at peri-infarct areas 24 h after administration (36 h after reperfusion). Scale bar, 50 µm. (D) Quantitative analysis (n = 5) showing the fluorescence intensity of Iba1 per cell corresponding to C. The sham groups were used as negative control. Data are expressed as mean ± SEM. *P < 0.05, **P < 0.01, ***P < 0.001 versus the PBS groups, and ^#^*P* < 0.05, ^##^*P* < 0.01 by One-way ANOVA.

Strategies to suppress inflammation hold great promise among the potential therapeutic approaches for ischemic stroke 3,4. Several proinflammatory cytokines, such as TNF-α, IL-1β, and IL-6, are responsible for infarct evolution and tissue injury 38. In the mouse MCAO/R model, the TNF-α, IL-1β, and IL-6 mRNA expression levels increased within 4-6 h of reperfusion and peaked at 18-24 h 34. To evaluate the anti-inflammation potential of RGD-EV_ReN_, these EVs were injected through the tail vein after 1 h of MCAO and 12 h of reperfusion and the mRNA or protein levels of the three proinflammatory cytokines in the lesion region were analyzed 12 h or 24 h later, respectively. Treatment with RGD-EV_ReN_ resulted in significantly strong suppression of all three cytokines, while EV_ReN_ or Scr-EV_ReN_ had minimal to no effect (**Figure 5A-B**).

Next, microglia activation, which is associated with neuroinflammation, was assessed. PBS, RGD-EV_293_, EV_ReN_, Scr-EV_ReN_, or RGD-EV_ReN_ were intravenously administered after 1 h of MCAO and 12 h of reperfusion. Twenty-four hours later, the brain slices were immunostained for Iba-1, a marker for microglia activation. Because microglia in the ischemic core are degenerated and fragmented, we examined the microglia in the peri-infarct area. In the sham group, microglia with thin ramified processes and small cellular bodies were observed in their resting form (**Figure 5C**). In contrast, MCAO/R induced strong staining of activated microglia with highly branched processes. Injection of RGD-EV_ReN_ resulted in a strong reduction in fluorescence intensity, as compared to EV_ReN_ or Scr-EV_ReN_ (**Figure 5D**). These results indicate that treatment with RGD-EV_ReN_ suppressed the inflammatory response after cerebral ischemia to a greater extent as compared to undecorated EVs due to their targeting ability resulting from RGD-4C peptide association. Finally, no obvious liver toxicity or tissue damage was observed in the RGD-EV_ReN_ treated mice compared with the control group (**Figure S8**).

### miRNAs enriched in EV_ReN_ inhibit MAPK pathway

**Figure 6:**
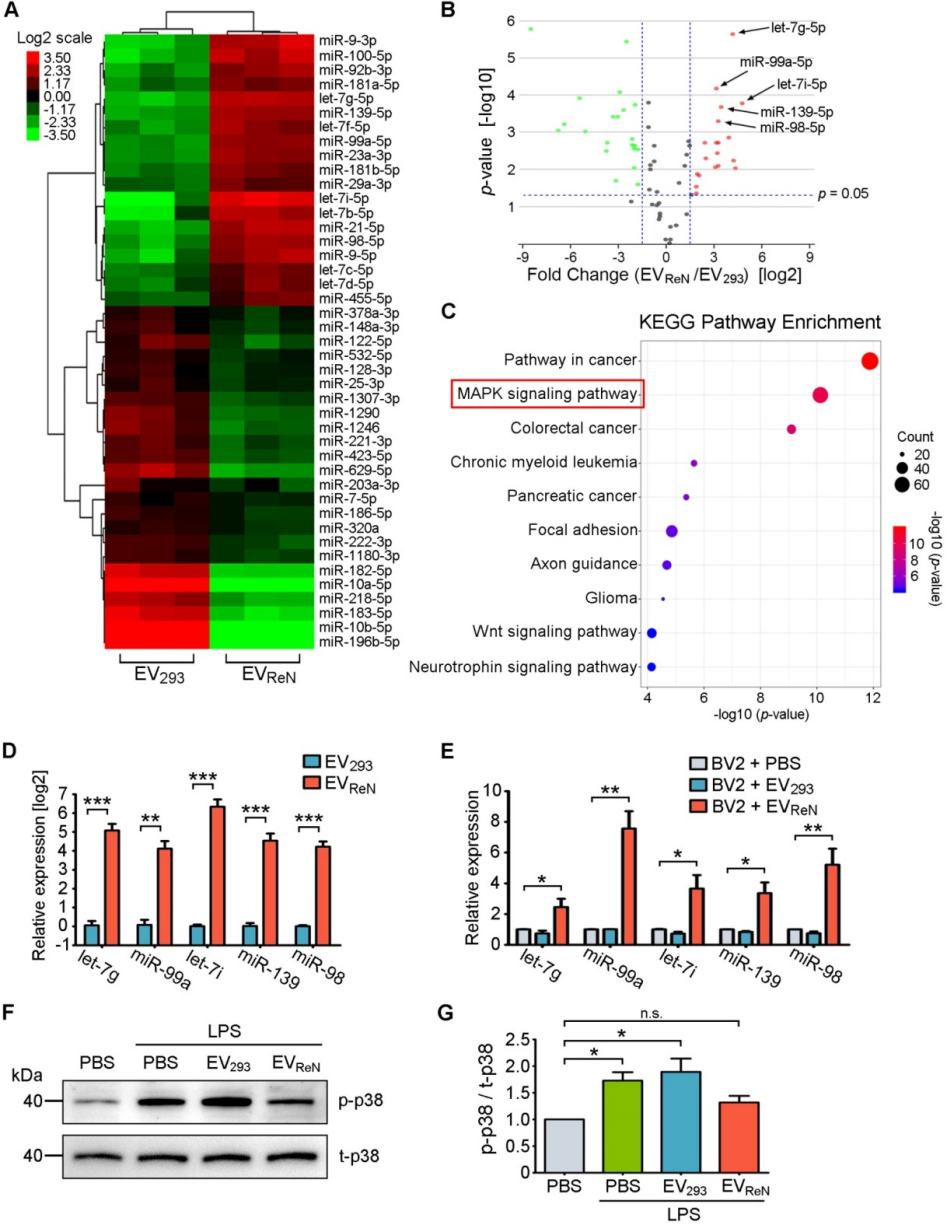
**miRNAs enriched in EV_ReN_ inhibit MAPK pathway.** (A) Clustering and Heatmap analysis (n = 3) of 43 differentially packaged miRNAs in EV_293_ and EV_ReN_ (TPM_average_ > 1000, fold change > 1.5 and *p* < 0.05). (B) Volcano plot shows the relation between the *p*-values and the fold changes. (C) KEGG pathway analysis of the target genes of 7 significantly up-regulated miRNAs in EV_ReN_. Top 10 enriched pathways are indicated. (D) RT-PCR analyses (n = 3) of top 5 differently packaged miRNAs in EV_293_ and EV_ReN_. cel-miR-39 as the external reference. (E) RT-PCR analyses (n = 3) of the miRNAs in BV2 cells were performed 24 h after the treatment of PBS, EV_293_ or EV_ReN_. U6 as the internal reference. (F) BV2 cells were pretreated with PBS, EV_293_ or EV_ReN_ for 24 h before LPS treatment. The expression levels of phosphorylated p38 (p-p38) and total p38 (t-p38) in the cells were determined by Western blot analysis 24 h after LPS treatment. (G) Quantification of p-p38 (n = 3) was normalized to t-p38 and presented as a relative change compared with the group without LPS treatment. Data are expressed as mean ± SEM. *P < 0.05; **P < 0.01; ***P < 0.001; n.s., no significance versus the first groups by One-way ANOVA.

To further define the underlying mechanism of the anti-inflammatory effect of EV_ReN_, differentially expressed miRNAs in EV_293_ and EV_ReN_ were identified by miRNA sequencing. Using TPM_average_ > 1000, 1.5-fold change and *p* < 0.05 as the threshold cutoff, 43 miRNAs were revealed to be significantly up- or down-regulated in EV_ReN_ compared with EV_293_ (**Figure 6A-B**). Subsequently, we performed target gene prediction and KEGG pathway analyses for top 7 up-regulated miRNAs including let-7g-5p, miR-99a-5p, let-7i-5p, miR-139-5p, miR-98-5p, miR-21-5p and let-7b-5p (**Figure 6C**). Interestingly, the predicted target genes were involved in MAPK, an inflammation related pathway. In addition, RT-PCR validated that the set of miRNAs were enriched in EV_ReN_ and up-regulated in BV2 cells receiving 24 h of EV_ReN_ treatment (**Figure 6D-E**). Finally, Western blot was used to confirm the effect of EV_ReN_ on LPS-stimulated MAPK signaling. A significant up-regulation of the phosphorylation level of p38, a key molecule in MAPK signaling pathway, was observed in LPS-treated BV2 cells, while EV_ReN_ suppressed the increase of phosphorylated p38 (**Figure 6F-G**). These results suggest that the EV_ReN_-mediated anti-inflammatory effect is highly dependent on MAPK pathway inhibition by miRNAs incorporated in EV_ReN_.

## Discussion

In this study, we first found that EV_ReN_ presented dose-dependent immunosuppressive activity on LPS-stimulated microglia, which are the primary mediators of the immune response after cerebral ischemia. As ReN cells are immortal cells that grow rapidly and retain normal karyotypes in culture after prolonged passage 25-27, they can act as robust donor cells for immunosuppressive EV production. To improve the targeting ability of EV_ReN_, RGD-4C, a doubly cyclizedpeptide binds to integrin α_v_β_3_ 200-fold more avidly than linear peptides, was used 39. High level of integrin α_v_β_3_ is found on the luminal surface of the endothelial cell only during angiogenesis 40,41. Thus, after cerebral ischemia, though present throughout the brain, integrin α_v_β_3_ preferentially expresses on the vascular surface in ischemic area.

We decorated the EV surface using an RGD-4C-C1C2 recombinant protein through a 15-min incubation. EVs are more enriched in PS than their donor cells and do not contain flippase to restrict PS in the inner leaflet of the membrane 32. The pull-down assay verified the association of RGD-C1C2 with EVs. Given that functional C1C2-fusion proteins can be prefabricated and stored in stock, the decoration process only requires a short incubation period. After decoration the RGD-EV_ReN_ presented an intact vesicle shape and normal size distribution. Although EVs express integrins on their membrane, no aggregation of RGD-EV_ReN_ was found. The reason may be that integrins often express in an inactive state in which they do not bind ligands 42. The idle state consists of a bent conformation that straightens to expose the binding site when the domain receives a signal from cytoplasm 43. Usually, this inside-out signal is mediated by PIP2-induced talin activation 44. It is hypothesized that the integrins on EVs received no active signaling during RGD-EV_ReN_ preparation in a PBS environment. Therefore, RGD do not interact with integrins on EVs with a high affinity in our system. In fact, RGD is a popular targeting ligand for surface modification of exosomes or EVs, however no aggregation phenomenon of RGD-decorated EV was reported previously 45,46.

A MCAO/R mouse model was used to evaluate the anti-inflammatory effects of RGD-EV_ReN_ *in vivo*. Our results showed significant accumulation of RGD-EV_ReN_ in the lesion region of the ischemic brain after intravenous administration. This targeting ability was indicated to be due to the affinity of the RGD-4C peptide to integrin α_v_β_3_ 47, which is expressed on cerebral vascular endothelial cells induced by ischemia. In addition, the anti-inflammatory effects of RGD-EV_ReN_ on the ischemic brain were confirmed by our data, indicating that the endogenous bioactivity of EVs was not impeded by RGD-C1C2 decoration or systemic administration. Therefore, RGD-EV_ReN_ acted as an effective targeting anti-inflammatory agent for cerebral ischemia therapy.

Recently, a large number of preclinical studies strongly suggested therapeutic potential of EVs in the cell-free treatment of inflammatory diseases 48. Specifically, mesenchymal stem cell (MSC)-sourced immunosuppressive factors are contained in MSC-derived EVs (EV_MSC_) 49. Following brain injury and ischemia, EV_MSC_ inhibited detrimental immune response by providing immunomodulatory factors in injured neurons and microglia 50. Remarkably, EV_MSC_ significantly suppressed activation of microglia, prevented reactive astrogliosis, and attenuated inflammation-induced neural degeneration 51. microRNAs enriched EV_MSC_ act as an important immunomodulatory factors, such as miR-21, miR-23a, miR-125b, and miR-145 52. Additionally, miR-146 and miR-21 may alter the phenotype, function, and viability of neural and immune cells in the therapy of inflammatory diseases 53. As an anti-inflammatory agent, EVs potentially avoid the limitations of stem/progenitor cell transplantation, such as risk of tumor formation, autoimmune responses, and ethical concerns. Importantly, immunomodulation and mediated by EV_MSC_ was either similar or even better than that by their parental MSCs 53. In this study, the anti-inflammatory effects of EV_ReN_ may be attributable to some modulatory molecules inherited from neural progenitor cells 27. According to previous literatures, primary neural progenitor cell-derived EVs (EV_NPC_) highly package miR-181, miR-26, miR-9 and the let-7 family which are predicted to inhibit microglia activation 6,54. In the present work, EV_ReN_ also exhibit distinct anti-inflammatory effects despite different miRNAs enriched. Given that ReN cells are cultured readily and grow rapidly, EV_ReN_ may be an alternative to EV_NPC_ for anti-inflammatory therapy. Furthermore, EVs have recently become known as an endogenous drug delivery system 55. Other RNAs (siRNAs and miRNAs), small therapeutic molecules, and proteins could be loaded into EV_ReN_ to improve their therapeutic effects in the future.

In summary, we found that ReN cells, a human neural progenitor cell line, were suitable for large-scale production of EVs with anti-inflammatory bioactivity. To improve the targeting ability of EV_ReN_, a recombinant protein containing C1C2 domains fused with the RGD-4C peptide was prepared and attached to the EV surface. The generated RGD-EV_ReN_ targeted the lesion region of the ischemic brain and suppressed poststroke inflammation. We believe that ReN cells are a robust source of therapeutic EVs and that this decoration method is suitable for the rapid production of functionalized EVs, thereby facilitating the clinical application of EVs.

## Supplementary Material

Supplementary figures.

## Abbreviations

PS: phosphatidylserine
MCAO: middle cerebral artery occlusion
MCAO/R: middle cerebral artery occlusion and reperfusion
EVs: extracellular vesicles
BBB: blood-brain barrier
ReN: ReNcell VM
EV_ReN_: ReN cell-derived EVs
RT-PCR: quantitative real-time PCR
ELISA: enzyme-linked immunosorbent assay
LPS: lipopolysaccharide
C1C2: C1 and C2 domains of lactadherin
MFGE8: milk fat globule-EGF factor 8 protein
RGD-4C peptide: ACDCRGDCFC
RGD-C1C2: a recombinant fusion protein of the RGD-4C peptide fused to C1C2
HA: hemagglutinin
RGD-EV_ReN_: RGD-C1C2-decorated EV_ReN_
NIRF: near-infrared fluorescence
NTA: nanoparticle tracking analysis
TEM: transmission electron microscopy
palm-tdTomato: tdTomato fused to palmitoylation signal
TNF-α: tumor necrosis factor-α
IL-1β: interleukin-1β
IL-6: interleukin-6
EV_293_: HEK293T cell-derived EVs
SS: signal sequence
Gluc: Gaussia luciferase
Gluc-TM: Gluc fused to the transmembrane domain of the platelet-derived growth factor receptor
Scr-C1C2: a scrambled ACDCRDGCFC peptide fused to C1C2
Scr-EV_ReN_: scrambled peptide-decorated EV_ReN_
TTC: 2, 3, 5-Triphenyltetrazolium chloride
Iba-1: ionized calcium binding adapter molecule 1
CTZ: coelenterazine
