# Supplementary material for: Targeted delivery of neural progenitor cell-derived extracellular vesicles for anti-inflammation after cerebral ischemia
Source: Theranostics. 2021 Apr 19;11(13):6507–21. doi: 10.7150/thno.56367 (PMC8120222; doi:10.7150/thno.56367)
Supplement: Supplementary file 1 — Supplementary figures. [file thnov11p6507s1.pdf]

Supplementary Materials

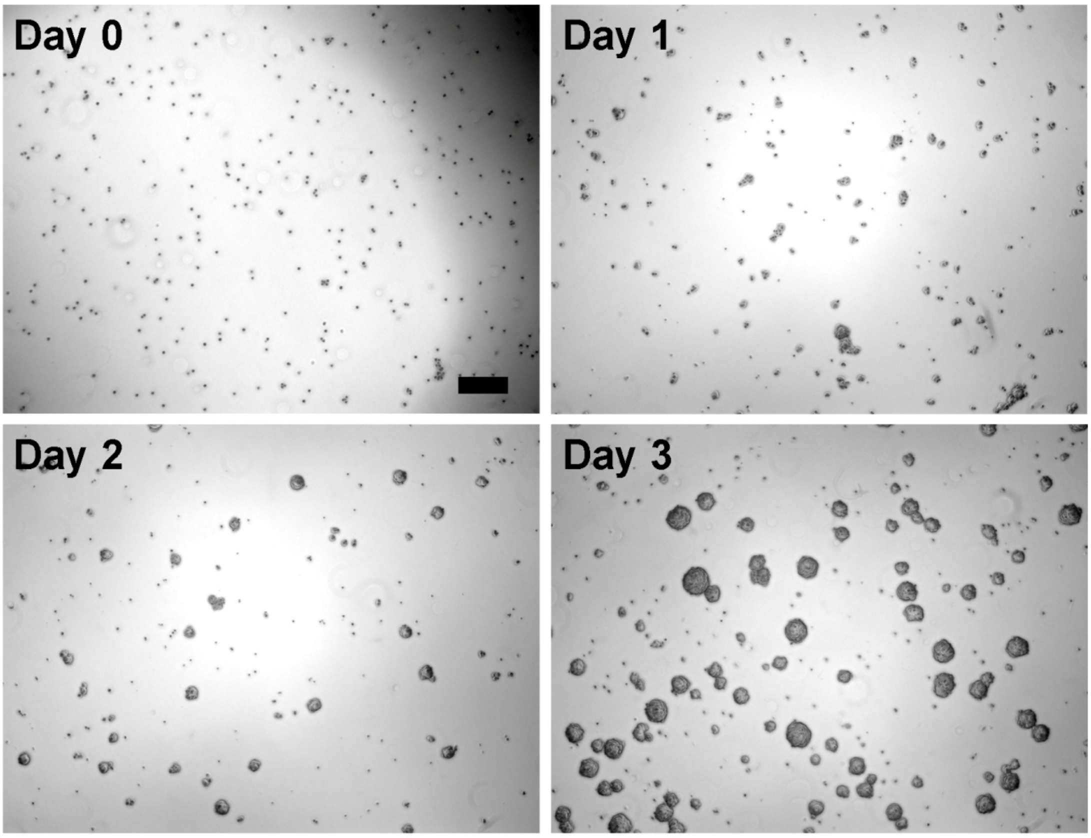

Figure S1. Bright field images of ReN cells cultured for 3 days. Scale bar, 200 μm.

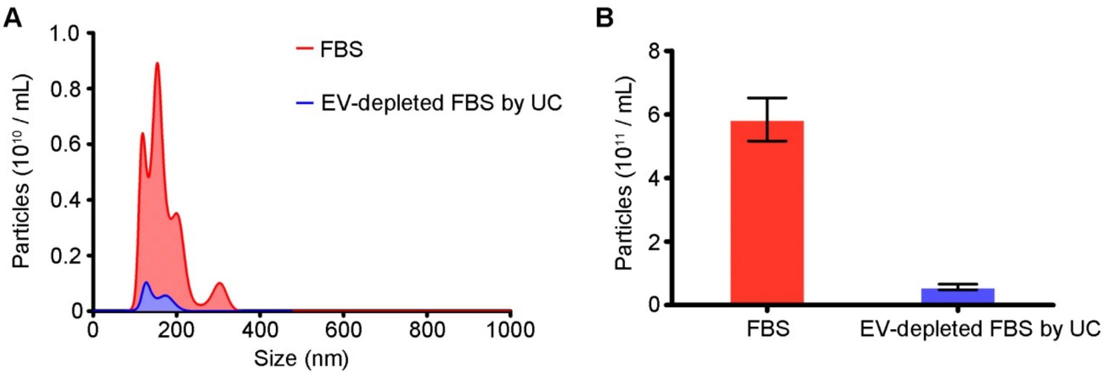

Figure S2. Size distributions (A) and particle concentrations (B) of FBS and EV-depleted FBS based on NTA measurements. UC: ultracentrifugation.

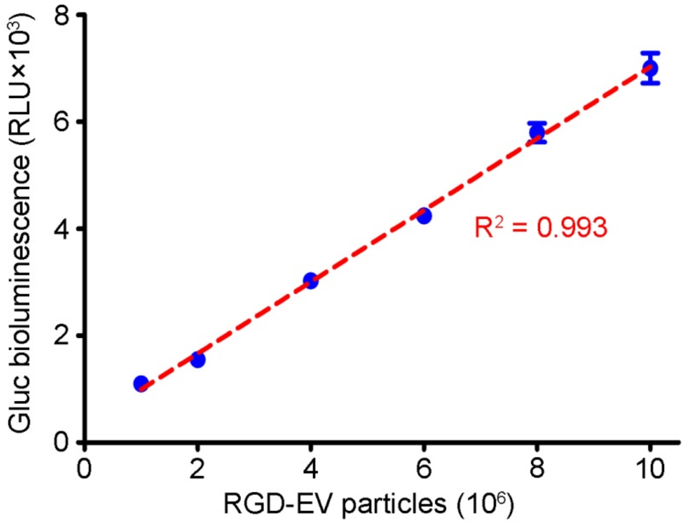

Figure S3. The linear relationship between Gluc activity in Gluc-labeled RGD-EV_{ReN} and the EV number. Blue dots indicate the average luminescence intensities of 1-10 × 10^6 Gluc-labeled RGD-EV_{ReN}. Red dash line is a linear fitting curve.

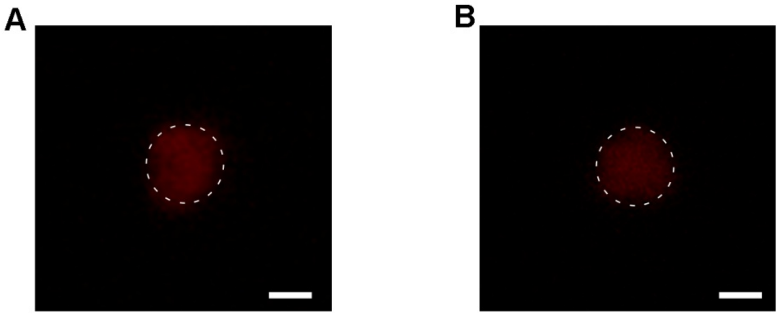

Figure S4. tdTomato-labeled EV_{ReN} were incubated with RGD-C1C2 following by a pull-down assay using anti-HA beads, and the fluorescence on the beads were detected by microscopy. Preincubation of the EV_{ReN} with lactadherin (A) or preincubation of the beads with the HA peptide (B) prevented the association of EV_{ReN} on the beads. The beads are around by dashed line. Scale bars, 1 μm.

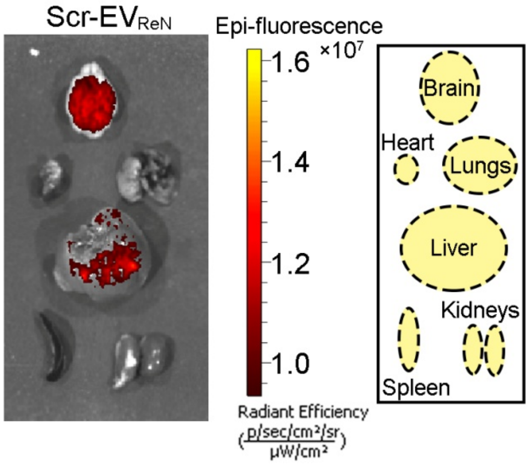

Figure S5. NIRF images of organs dissected from mice which received MCAO/R along with the administration of Cy5.5-labeled Scr-EV_{ReN}.

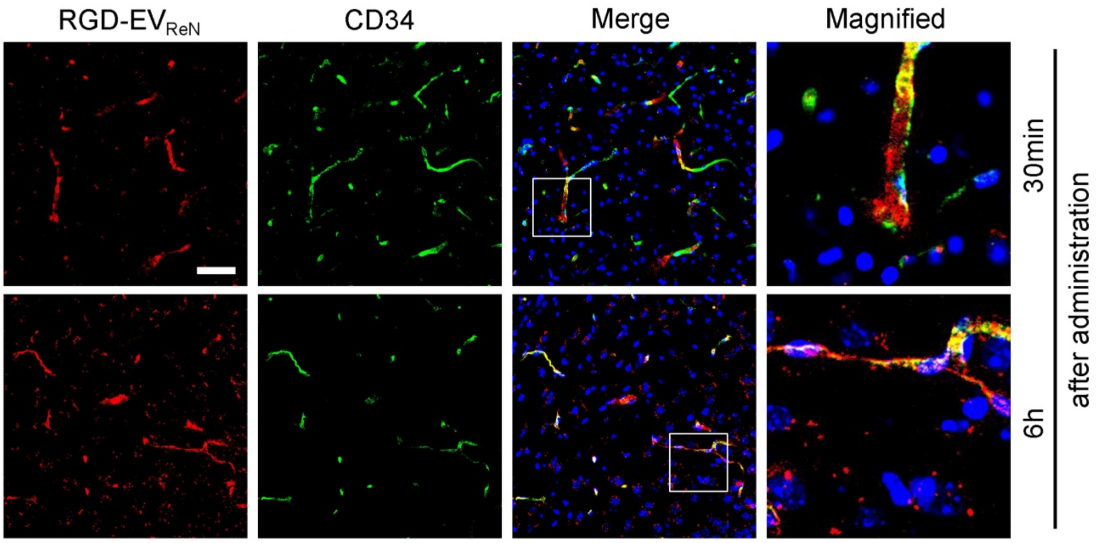

Figure S6. Co-labeled fluorescence images of CD34 (green) with RGD-EV$_{ReN}$ (red) in the lesion region 30 min after intravenous administration (12.5 h after reperfusion). Blue indicates nuclei. Scale bars, 50 μm. The images of 6 h after intravenous administration were borrowed from Figure 4D.

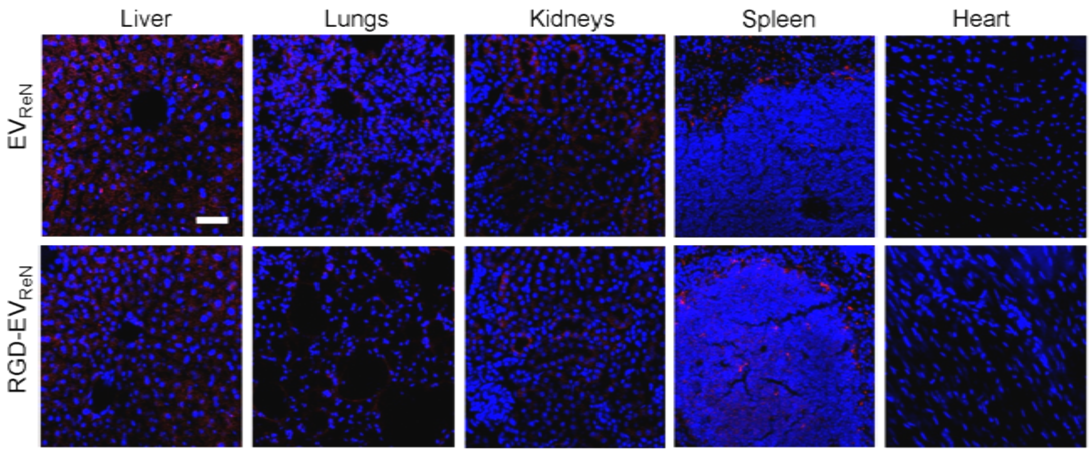

Figure S7. tdTomato-labeled EV_{ReN} or RGD-EV_{ReN} were administered intravenously on mice receiving 1 h of MCAO and 12 h of reperfusion. Fluorescence images of EVs (red) in the organs 24 h after administratoin (36 h after reperfusoin). Blue indicates nuclei. Scale bar, 50 µm.

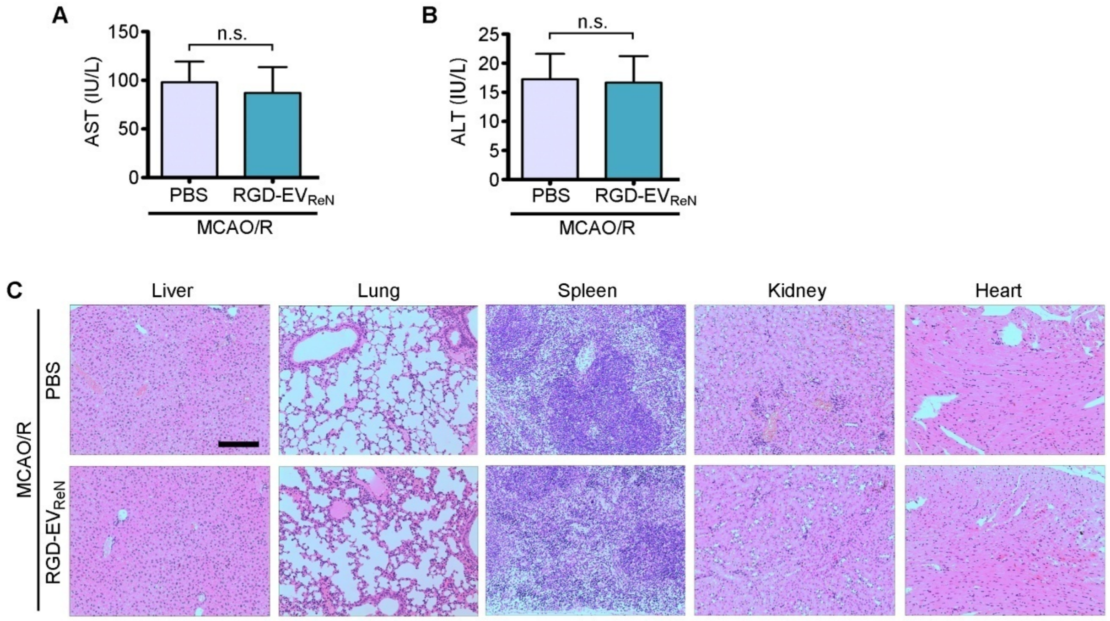

Figure S8. Safety evaluation for RGD-EV_{ReN} treatment. (A) The activity of aspartate aminotransferase (AST) and alanine aminotransferase (ALT) in the mice serum. (B) Hematoxylin and eosin (H&E) staining images for the organs in the mice. The mice were intravenously injected with PBS or 300 μg RGD-EV_{ReN} every day for 3 times. On the fourth day, the serum and organs of the mice were collected for analyzing. Scale bar, 200 μm.
